# Estimation and external validation of a new prognostic model for predicting recurrence-free survival for early breast cancer patients in the UK

**DOI:** 10.1038/sj.bjc.6605863

**Published:** 2010-09-07

**Authors:** H E Campbell, A M Gray, A L Harris, A H Briggs, M A Taylor

**Affiliations:** 1 Health Economics Research Centre, Department of Public Health, University of Oxford, Old Road Campus, Headington, Oxford OX3 7LF, UK; 2 Cancer Research UK Oxford Cancer Centre, Medical Oncology Unit, Churchill Hospital, Headington, Oxford OX3 7LJ, UK; 3 Health Economics Appraisal Team, Section of Public Health and Health Policy, University of Glasgow, 1 Lilybank Gardens, Glasgow G12 8RZ, UK

**Keywords:** prognostic model, early breast cancer, recurrence, survival analysis

## Abstract

**Background:**

We aimed to estimate and externally validate a new UK-specific prognostic model for predicting the long-term risk of a first recurrent event (local recurrence, metastatic recurrence, or second primary breast cancer) in women diagnosed with early breast cancer.

**Methods:**

Using data on the prognostic characteristics and outcomes of 1844 women treated for early breast cancer at the Churchill Hospital in Oxford, parametric regression-based survival analysis was used to estimate a prognostic model for recurrence-free survival. The model, which incorporated established prognostic factors, was externally validated using independent data. Its performance was compared with that of the Nottingham Prognostic Index (NPI) and Adjuvant! Online.

**Results:**

The number of positive axillary lymph nodes, tumour grade, tumour size and patient age were strong predictors of recurrence. Oestrogen receptor (ER) positivity was shown to afford a moderate protective effect. The model was able to separate patients into distinct prognostic groups, and predicted well at the patient level, mean Brier Accuracy Score=0.17 (s.e.=0.004) and overall C=0.745 (95% CI, 0.717–0.773). Its performance diminished only slightly when applied to a second independent data set. When compared with the NPI, the model was able to better discriminate between women with excellent and good prognoses, and it did not overestimate 10-year recurrence-free survival to the extent observed for Adjuvant! Online.

**Conclusion:**

The model estimated here predicts well at both the individual patient and group levels, and appears transportable to patients treated at other UK hospitals. Its parametric form permits long-term extrapolation giving it an advantage over other prognostic tools currently in use. A simple point scoring system and reference table allow 5-, 10-, and 15-year predictions from the model to be quickly and easily estimated. The model is also available to download as an interactive computer program.

It is acknowledged that the risk of recurrence and death in early breast cancer patients varies depending on an array of prognostic factors (Blamey *et al*, 1979; Haybittle *et al*, 1982; Walker, 2003; Chang and Hilsenbeck, 2004; Williams *et al*, 2006). Also established is that adjuvant systemic therapies (hormone therapy and chemotherapy) can prevent disease recurrence, yet their associated toxicities can adversely affect patient health-related quality of life (HRQoL; Chie *et al*, 2000; Conner-Spady *et al*, 2001; Sorensen *et al*, 2004; Early Breast Cancer Trialists' Collaborative Group, 2005a). These features of both disease and treatment, mean clinical decision making in early breast cancer is challenging. Predicting a patient's baseline risk is key in estimating the absolute benefit (survival net of any treatment-related decrement in HRQoL) likely to be afforded by treatment.

In the field of early breast cancer in the UK, statistical models and algorithms are often used to generate prognosis predictions. The Nottingham Prognostic Index (NPI), for example, which was developed using Cox proportional hazards modelling, has been widely used for this purpose (Blamey *et al*, 1979; Haybittle *et al*, 1982; Todd *et al*, 1987; Galea *et al*, 1992). Calculated as tumour stage plus tumour grade plus 0.2 × tumour size, a patient's NPI score classifies them into one of a number of prognostic groups, for which published survival curves are available and provide a point of reference for clinicians who wish to approximate prognosis.

Unfortunately, the heterogeneity present in early breast cancer means that within these NPI groups, the prognosis (and absolute effectiveness of treatment) for some patients will differ substantially from that of the group as a whole. In recognition of this, some clinicians in the UK have attempted to discriminate between patients within NPI prognostic groups. Examples include restricting chemotherapy to oestrogen receptor (ER)-negative patients with an NPI score ⩽3.4, or to women aged <50 years with an NPI score ⩽4.4 (Williams *et al*, 2006). Only recently has a more formal approach to obtain better baseline prognosis predictions using the NPI been proposed, with the publication of an algorithm for converting all individual NPI scores into 10-year survival percentages (Blamey *et al*, 2007).

Over the last decade, clinicians in the UK have made increasing use of Adjuvant! Online (http://www.adjuvantonline.com) for prognosis prediction and treatment benefit estimation (Ravdin *et al*, 2001; Olivotto *et al*, 2005; Siminoff *et al*, 2006). Adjuvant! is an internet-based program into which users can enter information on a patient's age, number of involved axillary lymph nodes, and the grade, size, and ER status of the primary tumour. The program returns predictions of 10-year overall survival, breast cancer-specific survival, and event (recurrence)-free survival, for each unique array of prognostic factor data entered. It, thus, has a greater discriminative ability than the NPI. In addition, Adjuvant! calculates the absolute survival benefit of any proposed adjuvant therapy by using treatment effect estimates from meta-analyses and randomized controlled trials to proportionately adjust its mortality and recurrence rates.

Adjuvant!'s estimates are not informed by prognostic modelling, but are derived instead from the observed 10-year survival experiences of over 30 000 women with invasive early breast cancer in the Surveillance, Epidemiology, and End-Results (SEER) tumour registry in the United States (Adjuvant! Online, 2005). Prognostic factor profiles entered into Adjuvant! are matched to those of women in the SEER registry. The mortality data from these matched cases (following certain adjustments) are then used as the basis of the program's survival predictions. Data on recurrence are not available from SEER, and so Adjuvant! estimates the risk of a recurrent event and thus event-free survival indirectly by inflating breast cancer mortality rates.

Despite its increasing acceptance in the UK, Adjuvant! has not been validated for use in this country. Indeed, a recent study assessing the performance of Adjuvant! in a cohort of UK early breast cancer patients showed the program's predictions to be optimistic – a finding most likely attributable to breast cancer mortality rates in the US being systematically lower than those in the UK (Cancer Information Section – International Agency for Research on Cancer, 2008; Campbell *et al*, 2009). Furthermore, and as Adjuvant!'s predictions are derived from observed 10-year outcome data rather than from a prognostic model, the program currently has no facility to predict long-term outcomes.

In this paper, we report on the estimation and external validation of a new UK-based parametric prognostic model for predicting long-term recurrence-free survival for early breast cancer patients. The model's performance is compared with that of the NPI and Adjuvant! Online, and a scoring algorithm and downloadable program to facilitate its use are presented.

## Materials and methods

### Data

The model was estimated using data collected on 1844 women with early invasive ductal carcinoma of the breast. These women were diagnosed consecutively and underwent surgery (77% breast conserving surgery, 23% mastectomy) at the Churchill Hospital, Oxford, between 1 January 1986 and 31 January 2001. Patient follow-up was till 31 January 2006 and was 89% complete (Clark *et al*, 2002). In all, 573 women suffered a first recurrent event (a local recurrence, a metastatic recurrence, or a second primary breast tumour). Median (range) time to a first recurrent event was 6.6 years (0.02–19.6 years). Columns 2 and 3 of Table 1 show the characteristics of the patient cohort.

Table 1 shows that with the exception of ER status, data were relatively complete. Even so, excluding women with any missing data would have reduced the sample size by approximately one quarter, and could have potentially introduced bias (if the women excluded were a non-random sample of the cohort). We therefore used multiple imputation (MI) to impute a number of values for each missing data point (van Buuren *et al*, 1999). Multiple imputation uses regression analysis to allow the correlations between the variables in the data set to be maintained when imputing. Through its prediction of ‘multiple’ values for each missing data point, the technique also accounts for the uncertainty in the imputation process *per se*. We performed MI in STATA (version 10; Stata Corp., College Station, TX, USA), imputing three values for each missing data point and in effect creating three separate data sets (Royston, 2004). A comparison of original and imputed data is presented in Table 1.

### Model estimation

**Figure 1:**
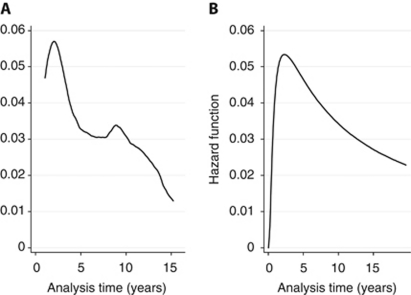
Recurrence hazard functions – for ‘average’ Churchill Hospital patient (**A**) and as predicted by the model (**B**). For comparability with **B**, which is evaluated at the mean of the covariates, the hazard contributions in **A** (estimated as the change in the Nelson–Aalen cumulative hazard between time *t*_i_ and time *t*_i−1,_ and smoothed by STATA's default kernel density function) were also calculated at the mean values of the model covariates using STATA's adjustfor(*varlist*) command.

Model estimation was also conducted in STATA. A parametric regression-based survival model was estimated on time from initial surgery to a first recurrent event or censoring (patients were censored when they died from causes unrelated to breast cancer without recurrence being first recorded (*n*=111/1844) or were lost to follow-up, again without any previous diagnosis of recurrence). Parametric models assume survival times and consequently the hazard function follow a particular distribution. Based on the hazard of recurrence for the average woman in the Churchill data set (Figure 1A), we considered models using log-normal, log-logistic, and gamma distributions. All three can model a hazard function which changes direction with time and are supported by the accelerated failure time (AFT) class of model (Bradburn *et al*, 2003). Accelerated failure time models differ from the more established proportional hazards (PH) models in that rather than estimating a baseline hazard function, AFT models instead estimate a baseline survival function. The estimated covariates are then multiplicative with respect to survival time. When exponentiated (transformed using the formula e^*x*^, where e=2.71828 and *x* is the coefficient value), the coefficients in an AFT model are termed as time ratios. A time ratio greater (less) than one indicates that a covariate increases (decreases) time to recurrence, stretching (shrinking) the baseline survivor function along the time axis.

In each of the MI data sets, time to a first recurrent event in days was regressed against the number of positive axillary lymph nodes (number), tumour grade (1, 2, or 3), tumour size (cm), ER status (positive or negative), and patient age (years). Continuous variables were retained on their original scales with the exception of ER, which was treated as dichotomous in nature. This was because different techniques for measuring ER (the ligand-binding assay (LBA) and the immunohistochemical (IHC) assay) were used during the course of the data collection. Although the assays used non-comparable measurement scales, both did use a cutoff score to indicate ER positivity/negativity. It was this dichotomous form of the variable that we entered into the model. In addition, also included were indicator variables for radiotherapy, and for adjuvant hormone therapy and chemotherapy. This would allow us to control for the effects of treatment received by patients in the data set (Table 1) so as to then have a prognostic model that could predict the baseline risk of recurrence in its absence (achieved by setting the indicator variables to zero).

For continuous variables, we investigated the functional form of the relationship between each variable and time to a first recurrent event using fractional polynomials (Royston and Altman, 1994). We also included in the model the following treatment/prognostic variable interactions: ER status and hormone therapy, age and adjuvant chemotherapy, and number of positive axillary lymph nodes and adjuvant chemotherapy.

Quantile–Quantile plots were used to determine the appropriateness of the AFT framework and Akaike's Information Criterion (AIC) to select between models estimated using the log-normal, log-logistic, and gamma distributions (Akaike, 1974; Bradburn *et al*, 2003). Across the three data sets created using MI there was agreement as to the type of distribution to use. For two of the covariates (number of positive axillary lymph nodes and tumour size), the data suggested a non-linear relationship with time to a recurrent event. Clinical opinion was used to confirm the plausibility of the indicated relationships before such transformations were made. Finally, there was agreement across the MI data sets on the significance of all modelled interactions.

The goodness of fit of each model was investigated by comparing each model's predicted hazard with the hazard for the average Churchill patient. In addition, the formal test proposed by Hosmer and Lemeshow was used (Hosmer and Lemeshow, 1999; Bradburn *et al*, 2003). The test compares the number of observed and predicted recurrences in risk groups within the data set at a fixed point in time. We selected a 5-year time point for the test, which was performed using 1589 patients. Recurrence status at 5 years was unobservable for the remaining 255 patients. A total of 94 women died from causes unrelated to breast cancer and 161 were lost to follow-up, before 5 years. All were recurrence free at the time of censoring.

Finally, the ‘micombine’ command in STATA was employed to generate one overall prognostic model by averaging across the individual models estimated on each of the three data sets.

### Model performance

The aggregated model's performance at 5 years was assessed separately in each of the three data sets generated using MI. The prognostic factors and treatments received by each patient were fed into the model and the resulting predictions were assessed with respect to calibration, accuracy, and discrimination (Mackillop and Quirt, 1997). Calibration was given by the ratio of predicted to observed 5-year recurrence-free survival across the cohort (*n*=1844). Assessments of accuracy and discrimination required information on recurrence status at 5 years and so were conducted only for patients for whom these data were available (*n*=1589). Brier accuracy scores (concerned with performance at the individual patient level) were calculated for each patient and then averaged (Brier, 1950). An accuracy score of 0 indicates that the model can perfectly forecast patient-level outcomes at 5 years. The worst score achievable is 1.

Overall C (equivalent to the area under the receiver operating characteristic curve measure routinely used to evaluate the performance of diagnostic tests), was used to assess the model's discriminative ability (Mackillop and Quirt, 1997). A score of 1 suggests that the model can perfectly discriminate between patients who will and will not experience a recurrence. A score of 0.5 indicates that the model has no discriminative ability.

To assess how well the model could predict outcomes on the basis of prognostic factors alone, we repeated the above analyses after setting the treatment indicator variables to zero. If reasonable performance levels could be retained (analysts have suggested that variation in prognosis is attributable to prognostic factors rather than any particular therapy), it would suggest that the model could be used to generate baseline prognosis predictions (Williams *et al*, 2006).

In addition, we calculated the prognostic index (PI) from the model for each patient – this is simply the sum of the constant, and the cross products of the coefficients and the specified prognostic factors. An approach described by Cox was used to investigate various ways of classifying women into five prognostic groups (the same number often used for the NPI) on the basis of this PI (Cox, 1957; Blamey, 1996). Cox suggested that depending on the distributional form of a random variable, groupings be constructed to minimise the loss of information (L) about differences between individuals. L is calculated as the weighted average of the variance of the PI across the chosen groups divided by the variance of the PI for the whole sample. Having identified the method of grouping which minimised L, we then graphed the Kaplan—Meier recurrence-free survival curves for each group and assessed the degree of separation between them.

### External validation

To determine the model's transferability, we performed an external validation exercise using data collected from a sub-sample of patients recruited to the Adjuvant Breast Cancer (ABC) Trial, an international study designed to assess whether adjuvant chemotherapy and/or ovarian suppression add to the benefits of 5 years of tamoxifen for early breast cancer patients (The Adjuvant Breast Cancer Trials Collaborative Group, 2007a, 2007b). The trial recruited 3854 women from 10 countries between 1992 and 2000. From this wider cohort, we selected UK patients with invasive ductal carcinoma and with the potential for at least 5 years of follow-up at 30 June 2004 when the trial data set was frozen for analysis. This sub-sample comprised 1789 patients from 70 UK hospitals, 588 of whom suffered a first recurrent event. Data were missing on time to event for two patients suffering local recurrences. These patients were excluded, leaving a cohort of 1787 patients with 586 first recurrent events. A small number of metastatic recurrences (*n*=27) that resulted in death were only discovered postmortem. For the purpose of this study, the date of recurrence for these patients was assumed to be 1 day before death. Recurrence status at 5 years was unobservable for 245/1787 patients. In all, 10 women had died from causes unrelated to breast cancer before 5 years and 235 women, recruited during 1998 and 1999 were awaiting a 5 year follow-up at the time the trial data set was frozen for analysis. All were recurrence free at the time of censoring.

Data on established markers were routinely recorded by UK clinicians participating in the trial. The level of missing data across these variables was low, with the exception of ER status, missing for 484/1787 (27%) women, the majority of whom were recruited between 1993 and 1995 when ER evaluation was often not integral to local practice. Multiple imputation (using data from ABC patients only) was used to impute three values for each missing data point.

The performance of the model in each of the resulting three MI data sets was examined using the techniques described above. For calibration, the whole cohort was used. For accuracy and discrimination, the 245 patients, for whom recurrence status at 5 years was unobservable, were excluded. Analyses were conducted on predictions made using the prognostic factor data and adjuvant therapies of ABC patients. Published treatment effects for radiotherapy, hormone therapy, and chemotherapy (here, cyclophosphamide, methotrexate, fluorouracil (CMF)) however were used instead of the treatment effects in the model, for which the indicator variables were set to zero (Early Breast Cancer Trialists' Collaborative Group, 2005a, 2005b). The published treatment effects were applied to each patient's baseline risk of recurrence as predicted using the five prognostic factors in the model. From this adjusted hazard, the corresponding recurrence-free survival function was then derived. We chose not to use the treatment effects from the model because they were estimated from observational data, which are known to suffer from selection biases in this regard. Indeed the potential exists for treatment effects estimated from observational data to contradict what is widely known and accepted (as discussed below). Also, much more robust estimates are now available from published meta-analyses. An assessment of how well the model predicted for ABC patients on the basis of their prognostic factors alone was also made.

### Comparison with other prognostic tools

Finally, we attempted to compare the performance of our prognostic model with that of the NPI and Adjuvant! Online. A direct comparison with the NPI proved difficult as the 10-year individualized prognosis predictions that one can generate using the new NPI algorithm are of breast cancer-specific survival rather than recurrence-free survival, as has been modelled here. We did, however, investigate the ability of frequently used NPI cut-point values to separate patients in the Churchill and ABC Trial data sets into five prognostic groups. Kaplan–Meier recurrence-free survival curves were computed for each NPI group and the degree of separation between the curves was compared with that seen when grouping patients on the basis of the PI from our model.

When comparing our prognostic model with Adjuvant! Online (which does report event (recurrence)-free survival), it was not possible to use ABC Trial data as only a handful of women had been followed up for 10 years. We therefore identified patients from the Churchill Hospital data set with the potential for at least 10 years of follow-up data (those treated between 1986 and 1996) and who met Adjuvant! eligibility criteria (they had undergone ‘definitive’ local treatment with either mastectomy, or breast conserving surgery and radiotherapy). This gave a sample of 1127 patients. Within this cohort were 252 patients for whom recurrence status at 10 years was unknown. A total of 100 of these women had died from causes unrelated to breast cancer before 10 years (all were recurrence free at the time of death), and 152 patients were recurrence free at their last contact, but had been lost to follow-up before 10 years. Excluding these patients left 875 women, 426 of whom had suffered a recurrent event. The prognostic factors and treatments of each of these patients were run through our prognostic model and Adjuvant! Online, and using the resulting predictions, we performed comparisons of calibration, accuracy, and discrimination. For consistency with Adjuvant!, which draws its treatment effect estimates for adjuvant therapy from the Early Breast Cancer Trialists' Collaborative Group (EBCTCG) overviews, we adjusted the model's baseline risk predictions using estimates from the same source (Early Breast Cancer Trialists' Collaborative Group, 2005a, 2005b).

In addition, we performed analyses comparing the performance of the two tools when using only prognostic factors to predict outcome. As Adjuvant!'s baseline prognosis predictions (i.e., its predictions ‘without adjuvant systemic treatment’) already appear to account for the impact of local radiotherapy, when performing this analysis and to ensure consistency between the two tools, predictions from the prognostic model had to be made by entering information not only on the prognostic factors of each patient, but also on radiotherapy received.

## Results

Table 2 presents the aggregated prognostic model. Based on the AIC statistics, a gamma distribution offered the most appropriate functional form. Quantile–Quantile plots (not shown) also confirmed the suitability of the AFT class of model.

**Figure 2:**
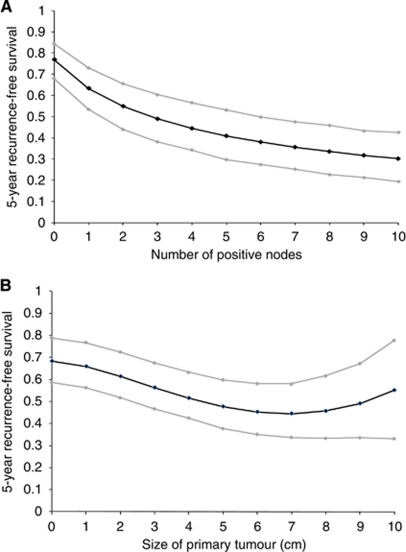
Graph showing for an average breast cancer patient, the probability of remaining recurrence free at 5 years (with 95% confidence intervals) as a function of the number of positive axillary lymph nodes (**A**) and size of primary tumour (**B**). Based on the Churchill data, this patient is ∼56.6 years of age, with 1.2 positive nodes, and a grade 2, ER-positive tumour 2.2 cm in diameter.

The regression coefficients in Table 2 have been exponentiated to give time ratios. As the number of positive axillary lymph nodes increased, recurrence-free survival decreased. This relationship was non-linear however, with a log transformation of the covariate offering a significantly better fit. As illustrated by Figure 2A, such a transformation implies that recurrence-free survival decreases with each additional positive node, but at a declining rather than a constant rate.

Tumour size was also an independent predictor of recurrence (see Table 2). Analyses suggested that the variable should be included in the model as follows: …*β* (tumoursize)^2^+*β* (tumoursize)^2^ × ln(tumoursize) … (where the *β*s are the estimated coefficients and ln is the natural logarithm). As shown in Figure 2B, this implies that for successively larger tumours, recurrence-free survival declines until a tumour diameter of ∼7 cm is reached. After this point, increasingly larger tumours appear to be associated with better prognoses.

Table 2 shows that the tumour grade was an independent predictor of shorter time to recurrence and that prognosis improved with increasing patient age. Women with ER-positive tumours also seemed to do slightly better than their ER-negative counterparts. The time ratios estimated from the actual treatments received showed (as one would expect) that for Churchill Hospital patients both radiotherapy and hormone therapy had a protective effect. Interpretation of the chemotherapy coefficient is difficult on account of the inclusion of various interaction terms within the model (chemotherapy and age, and chemotherapy and number of positive nodes). For patients identical in all respects other than the receipt of chemotherapy however, the model always predicted prognosis with treatment to be better than without it. This is expected, as chemotherapy was targeted at women in the Churchill data set with the poorest prognoses (i.e., those most likely to benefit).

In line with the published literature, coefficients estimated for the treatment interactions suggested that for women in the Churchill data set, ER status modified the effect of adjuvant hormone therapy (those with ER-positive tumours gaining the greatest benefit, as expected; Early Breast Cancer Trialists' Collaborative Group, 2005a). Age was also found to modify the effect of adjuvant chemotherapy however, the established inverse relationship (where treatment effectiveness declines with increasing age) was not observed. Instead, in the Churchill data set, chemotherapy appeared to be more effective for older women. Finally, the number of positive nodes modified the effect of adjuvant chemotherapy, with women with more positive nodes gaining more benefit.

### Goodness of fit

There was agreement as to the goodness of fit and performance of the model in all three data sets generated by the MI. Results are therefore presented for just one of these data sets (referred to from this point onwards as the Churchill Hospital data set). Figure 1B plots the predicted hazard function from the gamma model. Next to Figure 1A, it can be seen that the model's hazard function has the required uni-modal shape and peaks at a value not too dissimilar to the observed data and at around the same time. Results of the Hosmer and Lemeshow's test were also favourable, a *P*-value of *P*=0.112 suggesting no evidence for rejecting the null hypothesis that the model is of adequate fit.

### Model performance

Taking the ratio of model predicted (77.5%) to observed (76.8%) 5-year recurrence-free survival produced a calibration score of 101% (additional analyses by sub-groups are shown in Table A1 of the Web appendix). The mean Brier accuracy score was 0.15 (s.e.=0.005). Discrimination, assessed as overall C, was 0.764 (95% CI, 0.736–0.791). When prognostic factors only were used to estimate risk, predicted 5-year recurrence-free survival was 63.7%, giving a calibration score of 83%. The mean Brier accuracy score was 0.17 (s.e.=0.004), and overall C was 0.745 (95% CI, 0.717–0.773).

**Figure 3:**
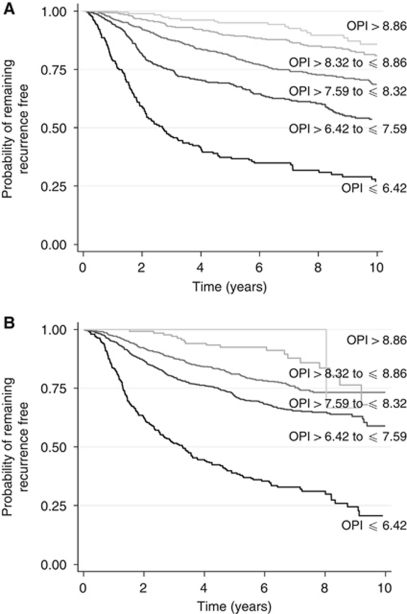
Kaplan–Meier recurrence-free survival curves for the Churchill Hospital (**A**) and ABC (**B**) data sets for five prognostic groups. (OPI cutoff values used to create groups are ⩽6.42, >6.42 to ⩽7.59, >7.59 to ⩽8.32, >8.32 to ⩽8.86, and >8.86).

Calculating the PI (hereafter referred to as the OPI – Oxford Prognostic Index) from the model for each patient in the Churchill Hospital data set generated a normally distributed variable ranging from 4.08 (worst prognosis) to 9.59 (best prognosis). For such a variable, Cox demonstrated that the loss of information from grouping (L) would be minimised by selecting cut points which place the following percentages of individuals into groups 1–5, respectively, 11, 23.7, 30.7, 23.7, 11%. Such grouping achieved a clear separation of the Kaplan–Meier recurrence-free survival curves (Figure 3A). The OPI cut points generated by grouping women into these five categories were ⩽6.42 (poor prognosis), >6.42, and ⩽7.59 (moderate II prognosis), >7.59 and ⩽8.32 (moderate I prognosis), >8.32 and ⩽8.86 (good prognosis), and >8.86 (excellent prognosis).

### External validation

External validation exercises were conducted in each of the three ABC data sets generated using MI. Findings were consistent across all three data sets and so are reported for one data set only (referred to from this point onwards as the ABC data set).

Predicted 5-year recurrence-free survival was 71.1% and observed 71.8%, giving a calibration score of 99% (additional analyses by sub-group are shown in Table A2 of the Web appendix). The mean Brier accuracy score was 0.19 (s.e.=0.005), and overall C was 0.720 (95% CI, 0.693–0.746). When using the model to forecast on the basis of prognostic factors only, predicted 5-year recurrence-free survival was 54.0%, and the resulting calibration score 75%. The mean Brier accuracy score was estimated to be 0.21 (s.e.=0.004), and overall C, 0.697 (95% CI, 0.669–0.726).

Finally, the OPI from the model was calculated for each patient in the ABC data set and the index cutoff values determined above were used to categorize patients into five groups. Figure 3B presents the Kaplan–Meier recurrence-free survival curves for each of these groups. The 5-year recurrence-free survival probabilities in each group were similar. In the Churchill and ABC data sets, respectively, they were 0.376 (95% CI, 0.306–0.445) and 0.391 (95% CI, 0.329–0.451) in the poor prognosis group, 0.688 (95% CI, 0.640–0.730) and 0.716 (95% CI, 0.682–0.745) in the moderate II prognosis group, 0.809 (95% CI, 0.772–0.840) and 0.810 (95% CI, 0.770–0.839) in the moderate I prognosis group, and 0.896 (95% CI, 0.861–0.922) and 0.925 (95% CI, 0.865–0.959) in the good prognosis group. A comparison for the excellent prognosis group is difficult on account of the small number of patients in the ABC data set falling into this category (*n*=13; Figure 3B).

### Comparison with other prognostic tools

**Figure 4:**
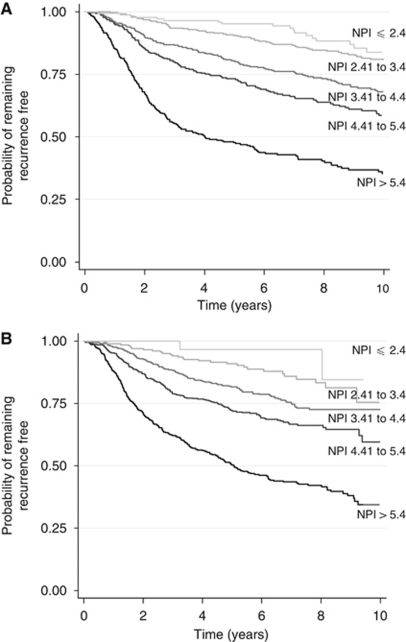
Kaplan–Meier recurrence-free survival curves for the Churchill Hospital (**A**) and ABC (**B**) data sets for five prognostic groups. (NPI cutoff values used to create groups are ⩽2.4, 2.41 to 3.4, 3.41 to 4.4, 4.41 to 5.4, and >5.4).

Figure 4A and B show for the Churchill and ABC data sets, respectively, Kaplan–Meier recurrence-free survival curves for each of five prognostic groups constructed using cut-point values for the NPI. Such values were: ⩽2.4 (excellent prognosis), 2.41–3.4 (good prognosis), 3.41–4.4 (moderate prognosis I), 4.41–5.4 (moderate prognosis II), and >5.4 (poor prognosis; Blamey, 1996). Although the NPI is able to discriminate between poor and moderate prognosis groups in the first few years following surgery, there is less separation between the recurrence-free survival curves of women classified as having good and excellent prognoses.

Observed 10-year recurrence-free survival across the 875 patients in the Churchill Hospital data set with complete follow-up data was 55.7%. Predicted 10-year recurrence-free survival estimates from the prognostic model and Adjuvant! Online were 61.3 and 67.3%, respectively, giving calibration ratios of 111 and 121%. Mean accuracy scores from the prognostic model and Adjuvant! Online were 0.22 (s.e.=0.005) and 0.22 (s.e.=0.007), respectively, and overall C, 0.706 (95% CI, 0.671–0.740) and 0.719 (95% CI, 0.686–0.754), respectively.

On the basis of prognostic factors only (yet also taking into account local treatment with radiotherapy), predicted 10-year recurrence-free survival estimates from the prognostic model and Adjuvant! Online were 51.4 and 58.7%, respectively, giving calibration ratios of 92 and 105%. Mean accuracy scores from the prognostic model and Adjuvant! Online were 0.21 (s.e.=0.005) and 0.21 (s.e.=0.006), respectively, and overall C, 0.720 (95% CI, 0.686–0.754) and 0.718 (95% CI, 0.684–0.752), respectively.

## Discussion

This paper reports on the development and validation of a new prognostic model for predicting recurrence-free survival in women with early breast cancer. Recurrence was selected as the dependant variable for two reasons. First, and although the aim of any treating clinician is unequivocally the prevention of breast cancer death, this cannot possibly be achieved without some consideration of an individual's risk of recurrence – the inevitable precursory event to breast cancer death. There would appear to be some benefit, therefore, from having access to a validated model, which can simulate the baseline risk of disease progression and the potential for adjuvant therapies to prevent this. Furthermore, alongside the more frequently encountered models predicting survival for breast cancer patients, those focussing on recurrence can help provide clinicians with a more complete picture of the entire disease process.

Second, and given the potential for post-recurrence survival to be influenced by a variety of factors (including the introduction of new therapies such as herceptin and taxanes for the treatment of advanced breast cancer), one could argue that first recurrence as an end point is likely to be more robust (once the impact of adjuvant therapy is taken into account) than breast cancer mortality.

We acknowledge that predictors of local and metastatic recurrences, and second primary breast recurrences may not necessarily be the same, however, the choice for our composite end point was informed by the work conducted by the EBCTCG and Ravdin and colleagues (Ravdin *et al*, 2001; Early Breast Cancer Trialists' Collaborative Group, 2005a). Both groups of researchers classify recurrence in this way and utilizing the same system allowed us to make use of the treatment effects reported in the EBCTCG Overview papers, and to perform comparisons between our prognostic model and Adjuvant! Online.

During the course of this study, we attempted to adhere to good practice guidelines for prognostic modelling (Harrell *et al*, 1996; Altman and Lyman, 1998; Altman and Royston, 2000; Bradburn *et al*, 2003; McShane *et al*, 2005). Before commencing the work, for example, checks were made to confirm that the study would be adequately powered, given the available data (Peduzzi *et al*, 1995; Harrell *et al*, 1996; Altman and Lyman, 1998). Also, we specifically utilized those modelling techniques, which would facilitate long-term patient-level prognosis prediction. We did not consider the Cox PH model, as its ‘distributional free’ form makes is difficult to describe the baseline hazard function (the key component required for patient level prediction) and to extrapolate outcomes beyond available observational data.

When selecting an appropriate distribution to model survival times, we followed published recommendations to graph the observed hazard and consider only those distributions that would give the same or a similar-shaped function (Bradburn *et al*, 2003). Additionally, so as to estimate a clinically meaningful model, a decision was made to force all five established prognostic factors into the model. We chose not to use stepwise variable selection techniques, which have been criticized for selecting variables for inclusion in a model based on statistical rather than clinical significance (Williams *et al*, 2006).

We retained continuous prognostic variables on their original scales and for each of these variables, we investigated the appropriateness of assuming a linear relationship with time to a first recurrent event. Transformations were indicated for the number of involved axillary lymph nodes and tumour size. The non-linear relationship seen between lymph nodes and recurrence has been observed previously, however, the relationship between tumour size and recurrence has not to our knowledge been reported elsewhere (it was though also observed in the ABC data; Sauerbrei and Royston, 1999; Sauerbrei *et al*, 1999). A number of researchers have investigated the relationship between tumour size and patient prognosis and found it to be non-linear, with the risk increasing with tumour size before eventually plateauing out (Jonat *et al*, 2002; Sauerbrei *et al*, 2003; Verschraegen *et al*, 2005). None of the patients in these studies, however, appear to have had tumours >5 cm in diameter, and so they provide no data on prognosis patterns of larger tumours. One possible explanation for the trend observed here is that patients in the Churchill data set who presented with the largest tumours may have had less proliferative and invasive disease, which had been slowly growing for many years before diagnosis. Indeed, a study looking at the effect of delays in diagnosis and treatment on outcomes in early breast cancer showed that women presenting early had poorer prognoses than women with far longer delays (Sainsbury *et al*, 1999). The authors suggested that more aggressive tumours that exhibit rapid growth or changes in size are more likely to prompt earlier presentation. Women with larger but very slow growing tumours which appear to change very little over time may be less likely to seek medical help.

As expected, higher grade tumours were associated with shorter recurrence-free survival times, and in line with the published literature, younger women had poorer prognoses than older women (de la Rochefordiere *et al*, 1993; Albain *et al*, 1994; Nixon *et al*, 1994). With respect to ER status, a factor for which the evidence of independent prognostic ability is mixed, the model suggested that the ER positivity afforded a small protective effect. The factor's clinical significance in routine practice is well established. Survey data from the UK show that ER status has been routinely used by clinicians alongside other established factors for patient level prognosis prediction (Williams *et al*, 2006). The widespread acceptance of Adjuvant! Online, a program in which ER status is a prognostic factor, provides a further indication of the importance placed on the factor by the clinical community (Ravdin *et al*, 2001). Any clinically credible prognostic tool would need to include ER status and so it was retained within the model.

The coefficient estimated for adjuvant hormone therapy exceeded one, suggesting that this treatment has some protective effect, regardless of ER status. Given the overwhelming evidence to show that hormone therapy is ineffective in ER-negative patients, this finding is interesting (Early Breast Cancer Trialists' Collaborative Group, 2005a). One possible explanation is that when using MI to deal with the issue of missing data, some women who were really ER positive, may have been imputed as ER negative. A further contributory factor may be the way in which a tumour's ER status is determined. Women whose assay results fall just short of the threshold value for positivity may still have some capacity to benefit from hormone therapy. Finally, and as with any diagnostic test, one would expect a number of false negative classifications.

Because the effects of adjuvant systemic therapy may be modified by certain prognostic characteristics, a number of treatment interaction terms were included in the model. The exponentiated coefficients for ER status and hormone therapy, and the number of positive axillary lymph nodes and chemotherapy, were in line with expectation. Based on the published literature however, which shows that older women receive less benefit from chemotherapy than younger women, one would have expected the time ratio for the age and chemotherapy interaction to have been <1 (Early Breast Cancer Trialists' Collaborative Group, 2005a). A closer inspection of the Churchill Hospital data set revealed that although the majority of women treated with chemotherapy received CMF, a slightly higher proportion of women over the age of 50 received anthracycline-based regimens. This is possibly an artefact of changes to clinical practice over time. Older women only started to receive chemotherapy routinely from the mid to late 1990s, when anthracycline use is likely to have been increasing. With evidence to suggest that these regimens are on average 11% more effective than CMF, this may well explain why the value for the time ratio for the age and chemotherapy interaction exceeded one (Early Breast Cancer Trialists' Collaborative Group, 2005a).

It is helpful to bear in mind that radiotherapy, hormone therapy, chemotherapy, and their interactions were included in the model not to estimate treatment effects (which are better estimated from large-scale meta-analyses). Rather the purpose was to adjust for the therapy administered to patients in order to have a model capable of generating predictions of recurrence-free survival on the basis of prognostic factors only. The increasingly widespread use of adjuvant systemic therapy is challenging for analysts developing new prognostic models. It is now no longer possible to study the relationship between prognostic factors and outcomes in cohorts of untreated breast cancer patients. Therapy is in routine use and so attempts should be made to isolate and adjust for its impact on prognosis.

In assessing the goodness of fit of the prognostic model, we compared the hazard functions estimated on the Churchill data and predicted by the model and saw the same uni-modal shape in both figures. The sharp decline in the hazard observed in the Churchill data at around 2 years, however, was not matched by the model, which showed a more gradual decline. One possible explanation is that the presentation of the observed hazard may be sensitive to the kernel densities and band widths used by STATA for hazard smoothing. Analyses performed using alternative values of these parameters, however, showed the impact on the function to be negligible, and despite the difference between Figure 1A and B, the formal test by Hosmer and Lemeshow still suggested that the model fitted the data well.

In addition to the challenges it poses for analysts estimating prognostic models, the widespread use of adjuvant therapy is equally an issue when assessing the performance and validity of these models. Indeed, in this study, the majority of patients in our development data set and all of the patients in the external validation data set had received some type of adjuvant therapy. When evaluating the performance of the model for Churchill hospital patients, assessments of calibration, accuracy, and discrimination were made on the basis of predictions generated with and without treatment. In each case, the model performed well at both group and individual patient levels, the accuracy and discriminative ability of the model decreasing only slightly when predictions were made on the basis of prognostic factors only. As discussed above, this perhaps reflects the suggestions of others that variability in prognosis is attributable largely to prognostic factors, rather than any particular therapy (Williams *et al*, 2006).

The real strength of this study lies in the external validation exercise performed. Patients in the ABC cohort differed from those in the Churchill Hospital cohort (ABC patients on average tended to have more positive nodes, and larger, higher grade tumours), however, the model still retained reasonable levels of prognostic and discriminative ability when applied to these patients, suggesting that it contains the main factors that explain variability in prognosis and is transferable to patients in other settings. In line with the analyses performed using the Churchill Hospital data, the performance of the model declined only slightly when predictions were generated using prognostic factors only.

When compared with the NPI, the OPI from the prognostic model appeared to be able to separate patients better (particularly those with excellent and good prognoses) in both the Churchill Hospital and ABC data sets into distinct prognostic groups. One must bear in mind, however, that the NPI was developed to model overall survival rather than recurrence-free survival as graphed here.

The findings of the comparison with Adjuvant! require further explanation. Although calibration ratios showed that Adjuvant! overestimated recurrence free survival for the cohort to a greater degree than the prognostic model, accuracy and discrimination statistics suggested both tools predicted as well as each other at the patient level. Further analyses of the accuracy scores generated during this exercise helped in explaining these findings. For women without recurrence at 10 years, accuracy scores appeared superior with Adjuvant! because its predictions of recurrence-free survival were generally higher than those of the prognostic model. For women diagnosed with recurrence however, the opposite was true – the prognostic model appeared to have superior accuracy as its predictions of recurrence-free survival tended to be lower than those of Adjuvant! When averaging across the whole cohort, the effect of this was to generate mean accuracy scores for both tools that were identical. Such findings are interesting and highlight the importance of considering the absolute predictions from prognostic models and tools in addition to performance-related statistics.

Although one could conclude that, on average, Adjuvant! overestimates recurrence-free survival compared with the prognostic model, one must take into consideration that it was necessary to perform this analysis using the Churchill Hospital data set, which could have afforded the prognostic model an unfair advantage. Further research using an independent data set with sufficiently long follow-up should be conducted to confirm these findings.

Although in the UK, the NPI and Adjuvant! Online are the two most widely used prognostic tools in early breast cancer today, in response to their apparent limitations, analysts continue to develop new prognostic models. Most recently, a group of researchers based in Cambridge, UK, published PREDICT, a new prognostic model that predicts overall and breast cancer-specific survival following surgery for invasive breast cancer (Wishart *et al*, 2010). PREDICT contains largely the same prognostic factors as the model presented in this paper and likewise has been externally validated with results suggesting good predictive and discriminative ability. Unlike the model presented here, PREDICT was estimated using Cox proportional hazards modelling (which precludes long-term extrapolation), and comparisons with Adjuvant! have yet to be published. PREDICT is not currently available for use by third parties.

This study has several limitations, the most obvious being the retrospective study design and the accompanying problem of missing data. We used MI to generate values for missing data points. This approach, which is favoured by analysts for its statistical integrity, has previously been used by researchers to handle the problem of missing data when conducting prognostic modelling studies (Clark and Altman, 2003). Evidence is also available to suggest that the technique remains valid under levels of missingness that exceed those observed here (Shafer and Olsen, 1998). Table 1 shows that with the exception of ER status, the imputed data is consistent with the original Churchill Hospital data for all prognostic and treatment variables. ER negativity was greater in the imputed data sets because the majority of patients with missing ER data were diagnosed in the late 1980s, before the introduction of the NHS breast screening programme and the consequent ability to diagnose greater numbers of less invasive, ER-positive tumours (Cancer Research UK, 2009).

A further and perhaps unavoidable limitation of this study and indeed prognostic modelling studies in general, is that the practices used to obtain prognostic factor data in a cohort have often been superseded by the time sufficient patient follow-up has accrued and the model is published. For example, over the 15-year period when Churchill Hospital patients in this study presented for surgery, techniques for assessing ER status changed (expensive LBAs were replaced by cheaper and simpler IHC assays), and a trend towards sampling increased numbers of axillary lymph nodes was also observed. Immunohistochemical assays have been shown to be an acceptable substitute for LBAs (similar levels of agreement have been observed between the two techniques) and so this change in practice is unlikely to impact on the prognostic modelling performed here. In contrast however, and if understaging was an issue in the Churchill Hospital data set (i.e., for women with fewer nodes sampled, the number of positive nodes was underestimated), then this could potentially bias the predictions from the prognostic model. To investigate this, we graphed Kaplan–Meier survival curves for women classified as having 0 positive nodes and 1–3 positive nodes on the basis of ⩽3, 4–6, 7–10, and >10 nodes sampled. We found no significant differences in recurrence-free survival according to the number of nodes sampled and therefore no evidence of under-staging.

The model reported here has been estimated and validated for use in predicting recurrence-free survival before adjuvant systemic therapy. For readers wishing to use the model, a simple scoring system, which can be used to predict a patient's probability of remaining disease-free at 5, 10, and 15 years post-surgery is available in the online Web-appendix. In addition, an interactive version of the model that allows the user to assess the potential impact of proposed adjuvant therapy is available to download from http://www.herc.ox.ac.uk.

Finally, although recurrence as the first sign of disease progression has been used as the dependant variable in the prognostic model presented here, clinicians and patients ultimately want to predict the likely impact of a disease and treatment on survival. To this end, we are currently taking steps to build on the work presented in this paper to develop a UK-specific prognosis-based lifetime disease progression model for early breast cancer. Following the success of Adjuvant! Online, this model will be computer-based and will facilitate the prediction of lifetime survival both with and without adjuvant therapy, as specified by the user. In addition, given the potential side effects of adjuvant therapy, the model will also adjust survival estimates for the impact of the disease and its treatments on patient HRQoL. In this respect, it will provide estimates of the net health benefit from adjuvant therapy, and so could potentially identify patients for whom treatment might on the whole be detrimental (i.e., the survival benefit is outweighed by the reduction in HRQoL from treatment). Given the increasing strain on already scarce health care resources, the model will also be programmed to compute lifetime costs. Interested users will then be able to determine the health economic implications associated with decisions to treat particular types of patient.

## Conclusion

This paper presents a new parametric prognostic model for predicting recurrence-free survival in UK patients with early breast cancer. The model has been shown to perform well and appears transportable to patients treated in other centres. Its parametric form also means that it is useful for extrapolating long-term predictions of the risk of recurrence in early breast cancer patients, and that it is therefore likely to be useful for analysts developing long-term disease progression models to assess the lifetime impact of new adjuvant therapies. We hope that the model presented here will prove complementary to similar models predicting breast cancer mortality in the sense that together they might provide a complete picture of the risk, first of disease progression and then ultimately of death.

## Figures and Tables

**Table 1:**
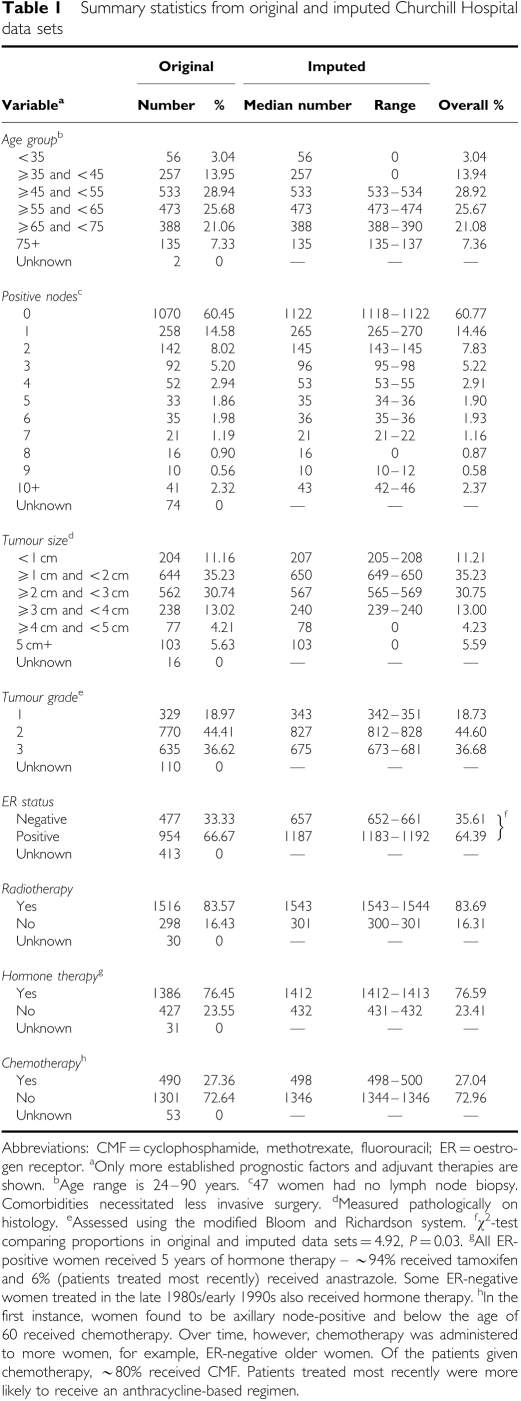
Summary statistics from original and imputed Churchill Hospital data sets

**Table 2:** Exponentiated coefficients (time ratios) from the final aggregated version of the prognostic model (estimated assuming survival times follow a gamma distribution)

	**Coefficient**	***P***	**95% CI**
ln (positive nodes)	0.402	<0.001	0.333–0.485
Tumour size^2^	0.898	<0.001	0.854–0.944
Tumour size^2^ × ln (tumour size)	1.045	<0.001	1.021–1.070
Tumour grade	0.647	<0.001	0.557–0.751
Age, years	1.015	0.005	1.004–1.026
Ercat	1.209	0.404	0.774–1.888
Adjrt	1.546	0.001	1.192–2.006
Adjhormones	1.230	0.234	0.875–1.730
Adjchemo	0.357	0.047	0.129–0.985
Ercat × adjhormones	1.226	0.481	0.696–2.160
Adjchemo × age	1.023	0.029	1.002–1.044
Ln (positive nodes) × adjchemo	1.418	0.015	1.070–1.878
Ancillary 1^a^	1.698	<0.001	1.572–1.835
Ancillary 2^a^	0.567	<0.001	0.411–0.782

Abbreviations: Adjchemo=adjuvant chemotherapy; Adjhormones=adjuvant hormone therapy; Adjrt=adjuvant radiotherapy; CI=confidence intervals; ER=oestrogen receptor; Ercat=ER status; ln=natural logarithm.

a Ancillary parameters 1 and 2 determine the shape and scale of the hazard function of the generalized gamma distribution. A literal interpretation of these parameter values is difficult however, on their original scales, they are useful for ruling out models with other functional forms nested within the gamma model. For example, if ancillary 1=ancillary 2=1, survival times follow an exponential distribution, and if ancillary 2=0 a log-normal model is appropriate.

## Supplementary Material

Supplementary Web appendix
