# Supplementary material for: Estimation and external validation of a new prognostic model for predicting recurrence-free survival for early breast cancer patients in the UK
Source: Br J Cancer. 2010 Sep 7;103(6):776–86. doi: 10.1038/sj.bjc.6605863 (PMC2966633; doi:10.1038/sj.bjc.6605863)
Supplement: Supplementary Web appendix [file 6605863x1.doc]

**Web Appendix**

**Table A1 Predicted and observed 5 year recurrence free survival and calibration ratios by subgroup (Churchill Hospital dataset)**

| **Group** | **Number** | **%** | **Predicted 5-year rfs** | **Observed 5-year rfs** | **Difference (pred-obs)** | **Calibration Ratio** |
| --- | --- | --- | --- | --- | --- | --- |
| *Nodal status*  Negative  Positive | 1118  726 | 60.63  39.37 | 85.02  65.82 | 84.61  64.82 | 0.41  1.0 | 100%  102% |
| *Tumour size*  ≤ 2cm  >2cm to ≤ 5cm  >5cm | 1037  736  71 | 56.24  39.91  3.85 | 84.04  70.99  48.46 | 84.93  68.26  44.75 | -0.89  2.73  3.71 | 99%  104%  108% |
| *Tumour grade*  I  II  III | 343  828  673 | 18.60  44.90  36.50 | 92.03  80.25  66.61 | 92.45  80.39  64.74 | -0.42  -0.14  1.87 | 100%  100%  103% |
| *ER status*  Negative  Positive | 661  1183 | 35.85  64.15 | 68.10  82.69 | 64.53  83.86 | 3.57  -1.17 | 106%  99% |
| *Age category*  ≤ 50 years  >50 years | 625  1219 | 33.89  66.11 | 72.96  79.77 | 73.39  78.65 | -0.43  1.12 | 99%  101% |

**Table A2 Predicted and observed 5 year recurrence free survival and calibration ratios by subgroup (ABC dataset)**

| **Group** | **Number** | **%** | **Predicted 5-year rfs** | **Observed 5-year rfs** | **Difference (pred-obs)** | **Calibration Ratio** |
| --- | --- | --- | --- | --- | --- | --- |
| *Nodal status*  Negative  Positive | 674  1113 | 37.72  62.28 | 78.86  66.40 | 80.68  67.11 | -1.82  -0.71 | 98%  99% |
| *Tumour size*  ≤ 2cm  >2cm to ≤ 5cm  >5cm | 954  772  61 | 53.39  43.20  3.41 | 74.94  67.52  56.15 | 79.54  64.36  58.62 | -4.6  3.16  -2.47 | 94%  105%  96% |
| *Tumour grade*  I  II  III | 196  772  819 | 10.97  43.20  45.83 | 82.32  73.89  65.77 | 87.47  76.33  64.73 | -5.15  -2.44  1.04 | 94%  97%  102% |
| *ER status*  Negative  Positive | 755  1032 | 42.25  57.75 | 61.44  78.15 | 63.49  78.61 | -2.05  -0.46 | 97%  99% |
| *Age category*  ≤ 50 years  >50 years | 1097  690 | 61.39  38.61 | 72.84  68.31 | 74.76  68.21 | -1.92  0.10 | 97%  100% |

**Construction of a point scoring system to facilitate easy use of the prognostic model**

To be useful in clinical practice, outcome predictions from prognostic models should be quick and simple to calculate. In reality however, multivariable regression models are often complex to implement, and when they contain several prognostic factors, it can be extremely tedious to work out the part of the regression equation that captures the individual’s risk profile, i.e. the sum of the cross products of the β coefficients with a patient’s values on the risk factors. Although computers offer the potential to ease such computational burden, another approach which uses a points-based scoring system to simplify the estimation of prognosis based upon complex statistical models, has also found favour with clinicians. Sullivan et al. have described this approach in detail, and so only a brief description is provided below.(Sullivan, Massaro & D'Agostino 2004)

In summary, the points system assigns integer points to each level of each risk factor in a prognostic model. By summing together the points that a patient scores on each risk factor and then looking up this total in a reference table which presents the model’s risk predictions that correspond to each point total, clinicians can quickly and easily determine a patient’s likely prognosis. The following steps are used when developing such a scoring system:

**Step 1** - Estimate the prognostic model.

**Step 2** -Organise each prognostic factor in the model into categories. For categorical variables no work is needed at this point, however continuous variables should be divided up into clinical meaningful categories, for example age might be split into five ten year age bands. Once categories are determined for each prognostic factor, work out a reference value (i.e. the mid-point) for each category. The reference value in an age category from 30 to 40 years for example, would be 35.

**Step 3** - For each risk factor, select the category which will be the base category, i.e. which will be assigned a zero in the scoring system. It is usual practice when doing this to use the category for each risk factor that is associated with the best prognosis.

**Step 4** - Determine how far, in terms of regression units, each reference value of each category is from the reference value in the base category for each prognostic factor.

**Step 5** - Define a constant for the points system, i.e. the number of regression units that will correspond to one point.

**Step 6** - Work out the points associated with each of the categories for each risk factor. This is done by dividing each of the values estimated in step 4, by the constant value determined in step 5.

**Step 7** – The point total for a patient with any combination of prognostic factors will **approximate** the linear predictor from the prognostic model, i.e. $\sum\beta X$. By substituting the point total (following adjustment for the base categories in the scoring system) into the prognostic model in place of the linear predictor, one can then use the model to predict the risk of an event. By working out the predicted level of risk associated with each achievable point total and then tabulating these data, one provides a reference table which clinicians can consult to determine the level of risk facing an individual with a particular point total.

A scoring system for the prognostic model was developed by following the various steps outlined above. Such systems, first developed by analysts working on the Framingham Heart Study, enable clinicians to quickly and easily convert a patient’s risk profile into a point score, which can then be ‘looked up’ in a reference table to obtain a corresponding prognosis prediction.(D'Agostino et al, 2000;Sullivan, Massaro & D'Agostino 2004) Table A3 below presents the estimated scoring system based upon the five established prognostic factors included in the model.

Table A3 Scoring system generated from prognostic model

| **Prognostic marker** | **Points** |
| --- | --- |
| ***Nodes***  0 Nodes  1 Positive Node  2 Positive Nodes  3 Positive Nodes  4-9 Positive Nodes  10+ Positive Nodes | 0  -9  -13  -17  -25  -33 |
| ***Tumour Size***  <1cm  1cm to <2cm  2cm to <3cm  3cm to <4cm  4cm to <5cm  >=5cm | 0  -2  -5  -8  -11  -13 |
| ***Tumour Grade***  1  2  3 | 0  -6  -12 |
| ***Patient Age***  <35 yrs  35 to <45 years  45 to <55 years  55 to <65 years  65 to <75 years  >75 years | -4  -2  0  2  4  6 |
| ***ER Status***  Positive  Negative | 0  -3 |

Table A4 below gives the model’s predicted probabilities of remaining disease free at 5, 10, and 15 years that correspond to various point totals. It is acknowledged that not all point totals listed in the table are achievable using the scoring system in table A3, however it is still helpful to see how the model’s predictions change with each point increment. Please note that the prognosis predictions presented in table A4 are based upon the five prognostic factors only (patient age, the number of positive axillary lymph nodes, and the size, grade and ER status of the primary tumour). They do not take into account the effects of any proposed radiotherapy, hormone therapy or chemotherapy. A separate interactive computer spreadsheet model, (downloadable from [http://www.herc.ox.ac.uk](http://www.herc.ox.ac.uk/)) is available which simulates the potential impact of various treatments (radiotherapy, hormone therapy, and chemotherapy) on the ‘baseline’ prognosis predictions shown in table A4.

Table A4 Reference table relating score totals (in bold) to probability of remaining recurrence free 5, 10, and 15 years following initial surgery

|  | **Probability of remaining recurrence free at...** | | | | |
| --- | --- | --- | --- | --- | --- |
| **Points Total** | **5 years** |  | **10 years** |  | **15 years** |
| **-65** | 0.0753 |  | 0.0428 |  | 0.0303 |
| **-64** | 0.0798 |  | 0.0455 |  | 0.0323 |
| **-63** | 0.0846 |  | 0.0484 |  | 0.0344 |
| **-62** | 0.0896 |  | 0.0515 |  | 0.0367 |
| **-61** | 0.0949 |  | 0.0547 |  | 0.0391 |
| **-60** | 0.1005 |  | 0.0582 |  | 0.0416 |
| **-59** | 0.1063 |  | 0.0618 |  | 0.0443 |
| **-58** | 0.1124 |  | 0.0656 |  | 0.0471 |
| **-57** | 0.1188 |  | 0.0696 |  | 0.0501 |
| **-56** | 0.1255 |  | 0.0739 |  | 0.0533 |
| **-55** | 0.1325 |  | 0.0784 |  | 0.0566 |
| **-54** | 0.1398 |  | 0.0831 |  | 0.0601 |
| **-53** | 0.1474 |  | 0.0880 |  | 0.0639 |
| **-52** | 0.1554 |  | 0.0932 |  | 0.0678 |
| **-51** | 0.1637 |  | 0.0987 |  | 0.0719 |
| **-50** | 0.1724 |  | 0.1044 |  | 0.0763 |
| **-49** | 0.1814 |  | 0.1104 |  | 0.0809 |
| **-48** | 0.1908 |  | 0.1167 |  | 0.0857 |
| **-47** | 0.2005 |  | 0.1233 |  | 0.0908 |
| **-46** | 0.2106 |  | 0.1302 |  | 0.0962 |
| **-45** | 0.2211 |  | 0.1374 |  | 0.1018 |
| **-44** | 0.2319 |  | 0.1449 |  | 0.1076 |
| **-43** | 0.2431 |  | 0.1528 |  | 0.1138 |
| **-42** | 0.2547 |  | 0.1610 |  | 0.1203 |
| **-41** | 0.2667 |  | 0.1696 |  | 0.1270 |
| **-40** | 0.2790 |  | 0.1785 |  | 0.1341 |
| **-39** | 0.2917 |  | 0.1877 |  | 0.1415 |
| **-38** | 0.3048 |  | 0.1973 |  | 0.1492 |
| **-37** | 0.3182 |  | 0.2073 |  | 0.1573 |
| **-36** | 0.3320 |  | 0.2177 |  | 0.1657 |
| **-35** | 0.3461 |  | 0.2284 |  | 0.1744 |
| **-34** | 0.3606 |  | 0.2395 |  | 0.1835 |
| **-33** | 0.3753 |  | 0.2510 |  | 0.1929 |
| **-32** | 0.3904 |  | 0.2628 |  | 0.2028 |
| **-31** | 0.4057 |  | 0.2750 |  | 0.2129 |
| **-30** | 0.4214 |  | 0.2876 |  | 0.2235 |
| **-29** | 0.4372 |  | 0.3006 |  | 0.2344 |
| **-28** | 0.4533 |  | 0.3139 |  | 0.2457 |
| **-27** | 0.4696 |  | 0.3275 |  | 0.2574 |
| **-26** | 0.4861 |  | 0.3416 |  | 0.2695 |
| **-25** | 0.5028 |  | 0.3559 |  | 0.2819 |
| **-24** | 0.5196 |  | 0.3706 |  | 0.2947 |
| **-23** | 0.5365 |  | 0.3855 |  | 0.3078 |
| **-22** | 0.5534 |  | 0.4008 |  | 0.3213 |
| **-21** | 0.5704 |  | 0.4163 |  | 0.3352 |
| **-20** | 0.5874 |  | 0.4321 |  | 0.3494 |
| **-19** | 0.6044 |  | 0.4482 |  | 0.3639 |
| **-18** | 0.6213 |  | 0.4644 |  | 0.3787 |
| **-17** | 0.6382 |  | 0.4808 |  | 0.3939 |
| **-16** | 0.6549 |  | 0.4975 |  | 0.4093 |
| **-15** | 0.6715 |  | 0.5142 |  | 0.4250 |
| **-14** | 0.6878 |  | 0.5311 |  | 0.4409 |
| **-13** | 0.7039 |  | 0.5480 |  | 0.4570 |
| **-12** | 0.7198 |  | 0.5650 |  | 0.4734 |
| **-11** | 0.7354 |  | 0.5820 |  | 0.4899 |
| **-10** | 0.7507 |  | 0.5990 |  | 0.5066 |
| **-9** | 0.7656 |  | 0.6159 |  | 0.5234 |
| **-8** | 0.7801 |  | 0.6328 |  | 0.5403 |
| **-7** | 0.7942 |  | 0.6496 |  | 0.5573 |
| **-6** | 0.8079 |  | 0.6662 |  | 0.5743 |
| **-5** | 0.8211 |  | 0.6826 |  | 0.5913 |
| **-4** | 0.8338 |  | 0.6988 |  | 0.6083 |
| **-3** | 0.8461 |  | 0.7148 |  | 0.6252 |
| **-2** | 0.8578 |  | 0.7305 |  | 0.6420 |
| **-1** | 0.8690 |  | 0.7458 |  | 0.6587 |
| **0** | 0.8797 |  | 0.7608 |  | 0.6752 |
| **1** | 0.8898 |  | 0.7755 |  | 0.6915 |
| **2** | 0.8994 |  | 0.7897 |  | 0.7076 |
| **3** | 0.9084 |  | 0.8035 |  | 0.7234 |
| **4** | 0.9169 |  | 0.8169 |  | 0.7389 |
| **5** | 0.9249 |  | 0.8298 |  | 0.7541 |
| **6** | 0.9323 |  | 0.8422 |  | 0.7689 |

**References**

D'Agostino, R. B., Russell, M. W., Huse, D. M., Ellison, R. C., Silbershatz, H., Wilson, P. W., & Hartz, S. C. (2000) Primary and subsequent coronary risk appraisal: new results from the Framingham study. *Am Heart J* **139:**272-281

Sullivan, L. M., Massaro, J. M., & D'Agostino, R. B., Sr. (2004) Presentation of multivariate data for clinical use: The Framingham Study risk score functions. *Stat Med* **23:**1631-1660, doi10.1002/sim.1742
